# Unravelling the clinical heterogeneity of undefined recurrent fever over time in the European registries on Autoinflammation

**DOI:** 10.1186/s12969-024-00987-z

**Published:** 2024-05-17

**Authors:** Y. Vyzhga, H. Wittkowski, V. Hentgen, S. Georgin-Lavialle, A. Theodoropoulou, S. Fuehner, M. Jesenak, J. Frenkel, E. Papadopoulou-Alataki, Jordi Anton, A. Nunzia Olivieri, J. Brunner, J. Sanchez, I. Koné-Paut, S. Fingerhutova, P. Pillet, U. Meinzer, R. Khubchandani, A. Jansson, J.-P. Haas, R. Berendes, T. Kallinich, G. Horneff, E. Lilienthal, R. Papa, D. Foell, E. Lainka, R. Caorsi, M. Gattorno, M. Hofer, AID-Net JIRcohortEurofever/PRINTO

**Affiliations:** 1 National Pirogov Memorial Medical University, Vinnytsya, Ukraine; 2 Department of Pediatric Rheumatology and Immunology, University Hospital Munster, Munster, Germany; 3 Department for Pediatrics, National Referral Centre of Auto-Inflammatory Diseases and Inflammatory Amyloidosis, - CEREMAIA, Versailles Hospital, Le Chesnay (Paris), France; 4 CEREMAIA (French Reference Center for Auto-Inflammatory Diseases and Inflammatory Amyloidosis), Kremlin-Bicêtre, France; 5 Department of Internal Medicine, Sorbonne University, Tenon Hospital (APHP), Paris, France; 6 Department of Pediatrics, Centre Hospitalier Universitaire Vaudois (CHUV), Lausanne, Switzerland; 7 Department of Peadiatrics and Adolescent Medicine, Jesenius Faculty of Medicine in Martin, Comenius University, University Hospital Martin, Martin, Slovakia; 8 Department of Pediatric Immunology and Rheumatology, Wilhelmina Kinderziekenhuis, Utrecht, Netherlands; 9 Department of Pediatrics, School of Medicine, Faculty of Health Sciences, Papageorgiou General Hospital, Fourth, Aristotle University of Thessaloniki, Thessaloniki, Greece; 10 Department of Pediatric Rheumatology, Hospital Sant Joan de Déu, Universitat de Barcelona. Institut de Recerca Sant Joan de Deu, Barcelona, Spain; 11 Dipartimento Della Donna del Bambino E Di Chirurgia Generale E Specialistica, Università Degli Studi Della Campania L.Vanvitelli, Naples, Italy; 12 Department of Pediatrics, Pediatric Rheumatology, Medical University Innsbruck, Innsbruck and Danube Private University Krems, Innsbruck, Austria; 13 Hospital Parc Taulí de Sabadell, Reumatologia Pediàtrica - Servei de Medicina Pediàtrica, Barcelona, Spain; 14 Department of Pediatric Rheumatology, National Referral Centre of Auto-Inflammatory Diseases and Inflammatory Amyloidosis, CEREMAIA, CHU de Biĉetre, APHP, University of Paris Sud, Le Kremlin Biĉetre, France; 15 Department of Paediatrics and Inherited Metabolic Disorders, Centre for Paediatric Rheumatology and Autoinflammatory Diseases 1st Faculty of Medicine, Charles University and General University Hospital, Prague, Czech Republic; 16 Pediatrics and Immunology, CHU Pellegrin, Bordeaux, France; 17 Department of General Paediatrics, Paediatric Infectious Disease and Internal Medicine, Assistance Publique-Hôpitaux de Paris, Robert Debré University Hospital, Université de Paris, Paris, France; 18 Department of Pediatrics, Jaslok Hospital, Mumbai, India; 19 Division of Pediatric Rheumatology and Immunology, Dr. Von Hauner Children’s Hospital, University Hospital Munich, Munich, Germany; 20 German Center for Paediatric and Adolescent Rheumatology, Garmisch-Partenkirchen, Germany; 21 Marien Children’s Hospital, Landshut, Germany; 22 German Rheumatism Research Center, Leibniz Institute Berlin Charité Universitätsmedizin Berlin, Paediatric Pneumology, Immunology and Critical Care Medicine and SPZ (Center for Chronically Sick Children), Berlin, Germany; 23 Department of Pediatrics, Asklepios Clinic Sankt Augustin GmbH, Sankt Augustin, Germany; 24 Department of Pediatrics, Ruhr University of Bochum, Bochum, Germany; 25 Centre for Autoinflammatory Diseases and Immunodeficiencies, IRCCS Istituto Giannina Gaslini, Genoa, Italy; 26 Department of Pediatric Rheumatology, University Children’s Hospital Essen, Essen, Germany

**Keywords:** Autoinflammatory diseases, PFAPA, SURF, Eurofever, JIR-cohort, AID-Net

## Abstract

**Background:**

Systemic autoinflammatory disorders (SAIDs) represent a growing spectrum of diseases characterized by dysregulation of the innate immune system. The most common pediatric autoinflammatory fever syndrome, Periodic Fever, Aphthous Stomatitis, Pharyngitis, Adenitis (PFAPA), has well defined clinical diagnostic criteria, but there is a subset of patients who do not meet these criteria and are classified as undefined autoinflammatory diseases (uAID). This project, endorsed by PRES, supported by the EMERGE fellowship program, aimed to analyze the evolution of symptoms in recurrent fevers without molecular diagnosis in the context of undifferentiated AIDs, focusing on PFAPA and syndrome of undifferentiated recurrent fever (SURF), using data from European AID registries.

**Methods:**

Data of patients with PFAPA, SURF and uSAID were collected from 3 registries including detailed epidemiological, demographic and clinical data, results of the genetic testing and additional laboratory investigations with retrospective application of the modified Marshall and PRINTO/Eurofever classification criteria on the cohort of PFAPA patients and preliminary SURF criteria on uSAID/SURF patients.

**Results:**

Clinical presentation of PFAPA is variable and some patients did not fit the conventional PFAPA criteria and exhibit different symptoms. Some patients did not meet the criteria for either PFAPA or SURF, highlighting the heterogeneity within these groups. The study also explored potential overlaps between PFAPA and SURF/uAID, revealing that some patients exhibited symptoms characteristic of both conditions, emphasizing the need for more precise classification criteria.

**Conclusions:**

Patients with recurrent fevers without molecular diagnoses represent a clinically heterogeneous group. Improved classification criteria are needed for both PFAPA and SURF/uAID to accurately identify and manage these patients, ultimately improving clinical outcomes.

**Supplementary Information:**

The online version contains supplementary material available at 10.1186/s12969-024-00987-z.

## Introduction

Systemic autoinflammatory disorders (SAIDs) are a growing spectrum of diseases in which the innate immune system is dysregulated. After the new concept of autoinflammation was first introduced in 1999, various previously known diseases have been attributed to a new genetic background or newly identified as monogenic autoinflammatory diseases. In addition, diseases with a clearly defined clinical picture, such as systemic juvenile idiopathic arthritis (SJIA), have been associated with an autoinflammatory pathophysiology without a monogenic background. Moreover, about half of the patients with recurrent inflammation do not fit the clinical picture of any well-defined SAIDs or do not have pathogenic mutations causing known hereditary SAIDs [1, 2].

Patients with recurrent fever without molecular diagnosis can often be diagnosed as Periodic Fever, Aphthous Stomatitis, Pharyngitis, Adenitis (PFAPA) syndrome, the most common pediatric autoinflammatory fever syndrome, with a high incidence in children up to 5 years of age. PFAPA may last up to adulthood with symptoms followed for years [3–7]. Clinical diagnostic criteria are well described for the disease [8].

Nevertheless, there is a number of patients that do not fulfil current diagnostic criteria of PFAPA and have variably been called as atypical PFAPA, undefined autoinflammatory disease or disorder, or syndrome of undifferentiated recurrent fever (SURF) [9]. Due to the rarity of SAIDs, clinical characteristics of such patients are not extensively described in the current literature [8, 10, 11].

It is still unclear, whether these patients represent autoinflammatory gene variants of known syndromes, complex genetic autoinflammatory disorders, or independent entities [9, 12]. But over the last few years, SURF definition was proposed as a heterogeneous group of autoinflammatory diseases characterized by the presence of self-limiting episodes of systemic inflammation with multiple clinical presentations, without confirmed molecular diagnosis. SURF is progressively more diagnosed in patients with recurrent fever after excluding the known hereditary recurrent fevers (HRF) and PFAPA syndrome [12].

Physicians face a variety of challenges when dealing with recurrent fever in children that can be caused by a number of different conditions, ranging from benign viral infections to more serious underlying medical conditions, as inborn errors of immunity, oncologic and autoimmune diseases. Awareness of autoinflammatory syndromes, in particular the diagnosis of PFAPA and SURF, as well as a better discrimination of both syndromes are essential for general paediatricians, paediatric rheumatologists, and even adult clinical immunologists.

The aim of this work was to analyse the evolution of symptoms in recurrent fevers without molecular diagnosis in the context of undifferentiated AIDs, with special regard to PFAPA and SURF, in three European AID-registries.

## Materials and methods

In a project endorsed by PReS, supported by the EMERGE fellowship program, and performed in line with the Metadata registry for the ERN RITA (MeRITA) project, we analysed the clinical characteristics of PFAPA, SURF and undefined systemic autoinflammatory disease (uSAID) patients in three registries, Eurofever, JIRcohort and AID-net, that collect and hold the information on SAIDs patients in Europe and in extra-European countries. In total, the 3 registries cover 7825 patients with different AIDs from 278 participating centres from different parts of the world with in-between country distribution as reported earlier [13]. AID-Net is a national registry involving 36 main pediatric rheumatology centers in Germany. JIR and Eurofever are multi-national cohorts, covering about 40 countries in total. Data collection in AID-Net was completed in 2018, but JIR and Eurofever continue active recruitment and follow-up on children previously included.

Data of patients with PFAPA, SURF and uSAID were collected from all 3 registries including detailed epidemiological, demographic and clinical data, results of the genetic testing and additional laboratory investigations, as well as treatment.

Patients of whom epidemiological and clinical information was available were included in this study. The diagnosis of the SAID – PFAPA, uSAID or SURF, was made by judgement of treating physician and exported from the registry. Extraction of the data and exclusion process was conducted separately for PFAPA and uSAID/SURF groups. Patients were excluded from further analysis if there was failure to complete information about clinical outcome of the disease. Patients carrying known pathogenic mutations, classifying them into group of other well-defined AIDs were excluded.

Requested clinical characteristics included disease manifestations, activity of the laboratory markers and genetic testing, if available; treatment was an optional part to be completed in registries. Clinical manifestations were reported by the corresponding physician in a different way through the registries but counted as “present” or “absent” within the study. Information on molecular genetic analyses was collected and classified according to pathogenicity. Only pathogenic or likely pathogenic variants were regarded to be an exclusion criterion.

Retrospectively, modified Marshall and PRINTO/Eurofever classification criteria were applied on the cohort of PFAPA patients [5, 6]. Preliminary case definition of SURF proposed by Papa et al. were applied for SURF and uSAID patients [12]. Evaluation of the extracted information was descriptive, more than comparative, as far as aggregated data from all three cohorts was not merged, yet analysed successively.

Categorical data were reported as absolute frequencies and percentages. Continuous data were described in terms of mean, SD, median, minimum and maximum, and 1st and 3rd quartiles (IQR). To compare dichotomous variables with interval or ordinal variables, the Mann–Whitney U test was performed. The threshold for statistical significance was *p* < 0.05.

## Results

### *Inclusion and clinical characteristic of PFAPA patients*

**Fig. 1:**
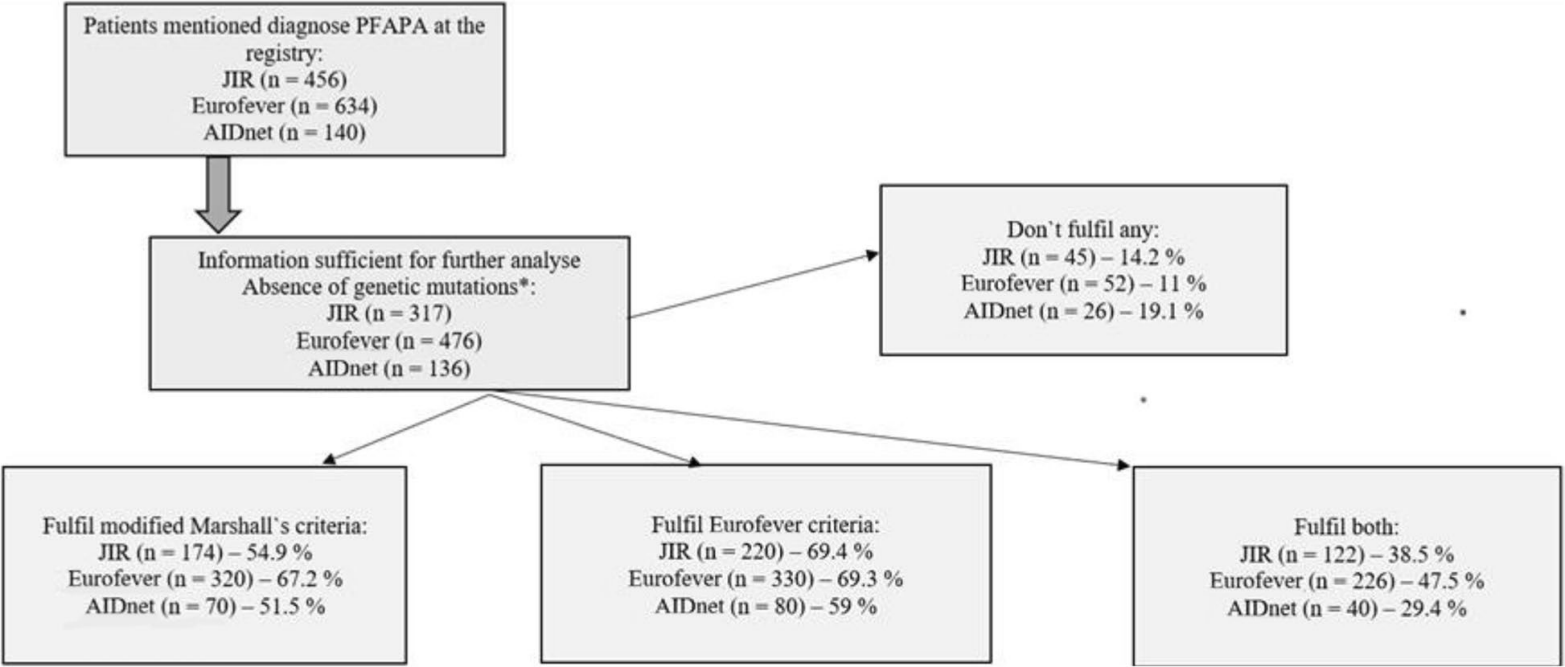
Flowchart of included patients with PFAPA

Out of the 1230 patients with PFAPA listed in Eurofever, JIR-cohort and AIDnet, we were able to identify 929 patients who fulfilled the primary requirements. These patients provided an adequate information pool (refer to Fig. 1).

The main primary objective of the study was to examine the evolution of PFAPA diagnosis over time, focusing on the period during which the disease was recorded in the registries. This was done by analysing the primary clinical symptoms present at the time of diagnosis, as outlined in Table 1.

**Table 1:** Evolution of the clinical symptoms of PFAPA patients recorded in the registries

Clinical symptoms at diagnosis	JIR-cohort (*n* = 317)	Eurofever (*n* = 476)	AID-Net (*n* = 136)
Fever			
< 2009	-	130/130 (100%)	-
2009 – 2012	2/2 (100%)	211/211 (100%)	23/23 (100%)
2013 – 2016	144/144 (100%)	63/63 (100%)	67/67 (100%)
2017 – 2020	171/171 (100%)	72/72 (100%)	46/46 (100%)
Pharyngitis			
< 2009	-	97/130 (74.6%)	-
2009 – 2012	2/2 (100%)	137/211 (64.9%)	13/23 (56.5%)
2013 – 2016	125/144 (86.8%)	41/63 (65.1%)	37/67 (55.2%)
2017 – 2020	133/171 (77.8%)	48/72 (66.7%)	23/46 (50%)
Cervical adenopathy			
< 2009	-	98/130 (75.4%)	-
2009 – 2012	2/2 (100%)	154/211 (72.9%)	18/23 (78.2%)
2013 – 2016	112/144 (77.8%)	45/63 (71.4%)	47/67 (70.1%)
2017 – 2020	113/171 (66.1%)*	56/72 (77.8%)	33/46 (71.7%)
Aphthous stomatitis			
< 2009	-	84/130 (64.6%)	-
2009 – 2012	2/2 (100%)	91/211 (43.1%)	11/23 (47.8%)
2013 – 2016	85/144 (59.0%)	30/63 (47.6%)	26/67 (38.8%)
2017 – 2020	62/171 (36.2%)*	35/72 (48.6%)*	20/46 (43.4%)

^*^p < 0.05

Number of the patients with reported features of PFAPA at the time of diagnosis

At the time of diagnosis, all PFAPA patients exhibited fever, but the presence of additional symptoms such as pharyngitis, cervical adenopathy, or aphthous stomatitis varied among them. The occurrence of pharyngitis did not show any significant difference over the duration of patient enrolment in all three registries. However, there was a tendency towards a decrease in the occurrence of both cervical adenopathy and aphthous stomatitis among PFAPA patients in the JIR cohort. A similar trend was observed in the occurrence of aphthous stomatitis reported among Eurofever patients with PFAPA. On the other hand, for AIDnet PFAPA patients, the frequencies of pharyngitis, cervical adenopathy and aphthous stomatitis remained stable across different periods of enrolment.

The diagnosis of PFAPA relies on clinical observations and can still be established even if not all symptoms are present. In our study, we used both the modified Marshall`s criteria and the Eurofever/PRINTO clinical classification criteria (see Supplementary Table 1) to assess the data.

Supplementary Table 1. Set of the clinical classification criteria used in the group of PFAPA patients among registries.

When the modified Marshall`s criteria were applied to patients from the JIR-cohort, Eurofever and AIDnet, 51.5% to 67.2% met them. However, the assessment of normal physical development and growth, which is included in the modified Marshall`s criteria, could not be considered due to limitations of the registries. In terms of completing the Eurofever/PRINTO clinical classification criteria, the percentage ranged from 59% to 69.4% for PFAPA patients, and 29% to 47.5% of them met both sets of criteria.

The clinical data set for patients with PFAPA, as shown in supplementary Table 2, demonstrates a similarity in the frequencies of the main signs across all registries and when applying both the Marshall`s and Eurofever/PRINTO criteria. The most common signs observed were fever, pharyngitis, and cervical adenopathy, while aphthous stomatitis was less common. The presence of a borderline age limit younger than 5 years old was found to be a limiting clinical sign in the groups of PFAPA patients who met both Marshall`s and Eurofever/PRINTO criteria.

However, for patients who met the Eurofever/PRINTO criteria, the absence of age limits resulted in a broader group with a higher average age at diagnosis (JIR-cohort: 4.43 years, Eurofever: 3.7 years and AIDnet: 4.2 years) and a longer diagnostic delay.

The application of both clinical classification criteria sets resulted in a more homogeneous group of responders, with an optimal diagnostic delay and a classical distribution of the frequencies of clinical signs: pharyngitis (76% to 92%), cervical adenopathy (70% to 85%), and aphthous stomatitis (40% to 52%) (Table 2).

**Table 2:** Clinical data of the patients completed both modified Marshall`s and Eurofever/PRINTO criteria

	JIR-cohort (*n* = 122)	Eurofever (*n* = 226)	AID-net (*n* = 40)
Age of diagnose years*	3.91 (0.81 – 4.99)	3.4 (1.2 – 4.83)	3.7 (0.75—5)
Boys/girls	68/54	117/109	21/19
Patients < 18 y.o. when diagnosed	122 (100%)	226 (100%)	40 (100%)
Fever > 38 C	122 (100%)	226 (100%)	40 (100%)
Duration of the episode, days^a^	4 (3—6)	3 (3—6)	3 (3—6)
Pharyngitis	113 (92.6%)	173 (76.5%)	31 (77.5%)
Cervical adenopathy	92 (75.4%)	159 (70.3%)	34 (85%)
Aphthous stomatitis	49 (40.1%)	106 (46.9%)	21 (52.5%)
Others			
Headache	22 (18%)	54 (23.9%)	15 (37.5%)
Diarrhoea	0	0	0
Rash	0	0	0
Arthritis	0	0	0

Patients with PFAPA selected from 3 registries and completed both – modified Marshalls and Eurofever/PRINTO criteria presented in current table, reflecting demography and relevant clinical information. ^a^Median, q1-q3

Our study reveals that a significant proportion, up to 19%, of the patients enrolled by centres as PFAPA do not meet the criteria either Marshall`s or Eurofever/PRINTO (supplementary Table 2). This group of patients has clinical features that differ from the classic PFAPA phenotype. Importantly, the incidence of classical signs such as pharyngitis, cervical adenopathy and aphthous stomatitis is relatively low. Instead, this subgroup of patients demonstrates a higher incidence of other symptoms, including diarrhoea (38% to 69%), skin rash (15% to 32%), arthritis and arthralgia (up to 8%). These findings suggest the presence of a distinct subgroup of patients labelled as PFAPA but presenting a different clinical profile.

### *Patients’ inclusion and clinicalcharacteristic of SURF and uSAID*

**Fig. 2:**
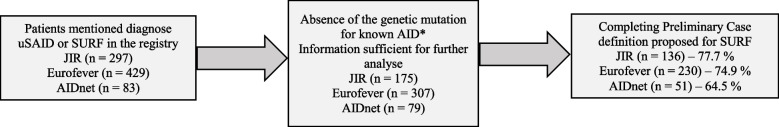
Flowchart of included patients with SURF/uSAID

Out of the 809 patients mentioned with the diagnosis of SURF or uSAID, we specifically chose 561 patients who had complete clinical information and did not have any pathogenic mutations associated with known autoinflammatory diseases. Our selection included patients with SURF from the JIR cohort and Eurofever, as well as those with uSAID from JIR-cohort, Eurofever and AIDnet. By applying the preliminary case definition for SURF [12], we further refined the group and ended up with 417 patients (136 from the JIR-cohort, 230 from Eurofever and 51 from AIDnet) for further analyse (Fig. 2).

Patients who had a confirmed diagnosis of uSAID or SURF and completed proposed case definition for SURF displayed a clinically homogeneous group characterized by a mean age at disease onset exceeding 6 years. Notably, a significant proportion of adult patients were also present, ranging from 10 to 23%. A common feature among these patients was the presence of periodic fever above 38˚ C, which was observed in 61% to 84% of cases. Additionally, other frequently reported symptoms included gastrointestinal involvement with abdominal pain (33% to 48%), diarrhoea (13% to 27%), and vomiting (5%—26%). Skin rash (21% to 53%), arthralgias (39% to 47%), and headache (27% to 31%) were also commonly observed. On the other hand, symptoms such as pharyngitis, cervical adenopathy, and aphthous stomatitis were less frequently observed among these patients (Table 3).

**Table 3:** Epidemiological and clinical data of the patients with uSAID/SURF according to the preliminary case definition for SURF (11)

	JIR-cohort (*n* = 175)		Eurofever (*n* = 307)		AID-net (*n* = 79)	
	uSAID/SURF fulfil SURF case definition and do not met PFAPA (*n* = 136)	uSAID/SURF do not met SURF case definition (*n* = 39)	uSAID/SURF fulfil SURF case definition and do not met PFAPA (*n* = 230)	uSAID/SURF do not met SURF case definition (*n* = 77)	uSAID/SURF fulfil SURF case definition and do not met PFAPA (*n* = 51)	uSAID/SURF do not met SURF case definition (*n* = 28)
Age of diagnose, years^a^	6.6 (0.9 – 63.4)	7.2 (2.9 – 29.8)	6.4 (3.1 – 9.2)	8.2 (3.6 – 21.3)	7.15 (1.0—18)	7.71 (2.41 – 18.7)
Diagnostic delay, years^a^	1.99 (0.1 – 47.9)	2.1 (0.7 – 6.61)	2.9 (0.6 – 11.2)	3.2 (1.9 – 7.4)	0.5 (0 – 17.5)	1.08 (0.1 – 17.5)
Boys/girls	67/69	17/22	119/111	44/33	33/18	17/11
Patients < 18 y.o. when diagnosed	104 (76.5%)	35 (89.7%)	196 (85.2%)	67 (87%)	46 (90.2%)	26 (92.8%)
Patients > 18 y.o. when diagnosed	32 (23.5%)	4 (10.3%)	34 (14.8%)	10 (13%)	5 (9.8%)	2 (7.1%)
Enrolled in time-period:						
•Under 2009	0	0	54 (23.5%)	20 (26%)	0	0
•2009 – 2012	0	0	91 (39.5%)	35 (45.4%)	26 (51%)	4
•2013 – 2016	46 (33.8%)	13 (33.3%)	41 (17.9%)	11 (14.3%)	19 (37.2%)	10
•2017 – 2020	90 (66.2%)	26 (66.7%)	44 (19.1%)	11 (14.3%)	6 (11.8%)	14
Fever > 38 C	136 (100%)	39 (100%)	230 (100%)	74 (96.1%)	51 (100%)	28 (100%)
-recurrent	83 (61%)	18 (46.1%)	N/A	N/A	43 (84.3%)	20 (71.4%)
-continued	9 (6.6%)	5 (12.8%)	N/A	N/A	8 (15.7%)	5 (17.8%)
Duration of the episode, days	4 (3—6)	2 (1—8)	4 (3—6)	2 (1—10)	4 (1—20)	2 (1—10)
Regular/ periodic attacks	59 (43.4%)	17 (43.6%)	88 (38.3%)	31 (40.2%)	N/A	N/A
Irregular/ non periodic attacks	56 (41.2%)	16 (41%)	123 (53.5%)	46 (59.7%)	N/A	N/A
Generalized lymphadenopathy	4 (2.9%)	1 (2.5%)	18 (7.8%)	4 (5.2%)	2 (3.9%)	2 (7.1%)
Cervical adenopathy	43 (31.6%)	11 (28.2%)	81 (35.2%)	25 (32.5%)	10 (19.6%)	4 (14.3%)
Muco-cutaneous signs						
•Aphthous stomatitis	32 (23.5%)	6 (15.4%)	64 (27.8%)	17 (22.1%)	4 (7.8%)	2 (7.1%)
•Rash	31 (22.8%)	6 (15.4%)	48 (21%)	14 (18.2%)	27 (53%)	13 (46.4%)
•Periorbital edema	3 (2.2%)	0	2 (0.1%)	0	2 (3.9%)	0
Pharyngitis	40 (29.4%)	6 (15.4%)	54 (23.5%)	10 (13%)	7 (13.7%)	0
Gastrointestinal involvement						
•Abdominal pain/fevers	63 (46.3%)	20 (51.3%)	112 (48.7%)	38 (49.3%)	17 (33.3%)	10 (35.7%)
•Diarrhoea	31 (22.8%)	8 (20.5%)	64 (27.8%)	12 (15.6%)	7 (13.7%)	4 (14.3%)
•Vomiting	36 (26.5%)	14 (35.9%)	52 (22.6%)	22 (29.8%)	3 (5.9%)	3 (10.7%)
Muscle-skeletal involvement						
Arthritis	10 (7.3%)	2 (5.1%)	13 (5.6%)	3 (3.9%)	8 (15.7%)	5 (17.8%)
Arthralgia	64 (47%)	33 (84.6%)	110 (47.8%)	42 (54.5%)	20 (39.2%)	12 (42.8%)
Myalgia	4 (2.9%)	3 (7.7%)	24 (10.4%)	11 (14.2%)	8 (15.7%)	5 (17.8%)
Morning stiffness	9 (6.6%)	1 (2.6%)	11 (4.7%)	3 (3.9%)	5 (9.8%)	0
Other manifestations						
Conjunctivitis	4 (2.9%)	0	6 (2.6%)	0	0	0
Chest pain	16 (11.7%)	5 (12.8%)	37 (16.1%)	11 (14.3%)	1 (1.9%)	1 (3.6%)
Headache	41 (30.1%)	11 (28.2%)	72 (31.3%)	17 (22.1%)	14 (27.4%)	8 (28.6%)
Hepatomegaly	3 (2.2%)	0	5 (2.2%)	1 (1.3%)	2 (3.9%)	1 (3.6%)
Splenomegaly	2 (1.5%)	0	3 (1.3%)	0	8 (15.6%)	3 (10.7%)
Pleuritis	3 (2.2%)	0	0	0	7 (13.7%)	1 (3.6%)
Pericarditis	6 (4.4%)	0	0	0	6 (11.7%)	2 (7.1%)
Other manifestations						
Conjunctivitis	4 (2.9%)	0	6 (2.6%)	0	0	0
Chest pain	16 (11.7%)	5 (12.8%)	37 (16.1%)	11 (14.3%)	1 (1.9%)	1 (3.6%)
Headache	41 (30.1%)	11 (28.2%)	72 (31.3%)	17 (22.1%)	14 (27.4%)	8 (28.6%)
Hepatomegaly	3 (2.2%)	0	5 (2.2%)	1 (1.3%)	2 (3.9%)	1 (3.6%)
Splenomegaly	2 (1.5%)	0	3 (1.3%)	0	8 (15.6%)	3 (10.7%)
Pleuritis	3 (2.2%)	0	0	0	7 (13.7%)	1 (3.6%)
Pericarditis	6 (4.4%)	0	0	0	6 (11.7%)	2 (7.1%)

^a^Median, q1-q3

Reflects information about uSAID/SURF patients who were initially selected from the general cohort and completed preliminary SURF criteria and did not met PFAPA criteria (column 1 for registry set) and utility of the data coming from the uSAID/SURF who did not fulfil preliminary SURF criteria (column 2 for registry set)

As expected, a subgroup of patients was listed with uSAID in the registries who did not fulfil the preliminary case definition for SURF. This subgroup consisted of 77 patients (25.1%) from Eurofever, 28 patients (35.4%) from AIDnet and 39 patients (22.3%) from JIR-cohort. These patients were 7 years old or older and exhibited typical periodic fever above 38˚ C, along with a wide range of gastrointestinal and musculoskeletal involvement. Classical signs such as pharyngitis and cervical adenopathy were rarely observed in this subgroup.

### *Overlap between PFAPA and uSAID/SURF*

Given the similarities in clinical signs observed between PFAPA, SURF and uSAID, we suspected the possibility of an overlap between these conditions. To explore this further, we applied the preliminary case definition for SURF [12] to the group of PFAPA patients and the PFAPA Eurofever/PRINTO classification criteria to the uSAID/SURF patients (refer to Table 4). This allowed us to investigate potential similarities and overlaps in the clinical profiles of these patients’ groups and gain insights into the relationship between these conditions.

**Table 4:** Clinical features of PFAPA patients fulfilling preliminary case definition for SURF and uSAID/SURF patients fulfilling Eurofever PFAPA criteria

	JIR-cohort		Eurofever		AID-net	
	PFAPA fulfils SURF (*n* = 54)	uSAID/SURF fulfils PFAPA (*n* = 66)	PFAPA fulfils SURF (*n* = 39)	uSAID/SURF fulfils PFAPA (*n* = 82)	PFAPA fulfils SURF (*n* = 16)	uSAID/SURF fulfils PFAPA (*n* = 21)
Age of diagnose, years^a^	5.11 (0.97 – 22.6)	5.75 (1.1 – 28.4)	3.9 (2.1 – 17.6)	4.35 (2.3 – 26.9)	4.58 (1.6 – 10.2)	6.58 (1.7 – 13.2)
Diagnostic delay, years^a^	2.87 (0.1 – 17.1)	1.55 (0.1 – 12.9)	2.1 (0.8 – 10.9)	2.38 (0.6 – 11.9)	2.83 (0.2 – 8.4)	0.62 (0.2 – 3.4)
Boys/girls	27/27	35/31	21/18	39/43	5/11	9/12
Patients < 18 y.o. when diagnosed	52 (96.3%)	61 (92.4%)	39 (100%)	71 (86.6%)	16 (100%)	21 (100%)
Patients > 18 y.o. when diagnosed	2 (3.7%)	5 (7.6%)	0	11 (13.4%)	0	0
Enrolled in time- period:						
•Under2009	0	0	17 (43.6%)	15 (18.3%)	0	0
•2009 – 2012	0	0	11 (28.2%)	28 (34.1%)	6 (37.5%)	7 (33.3%)
•2013 – 2016	17 (31.5%)	27 (40.9%)	3 (7.7%)	15 (18.3%)	9 (56.2%)	9 (42.8%)
•2017 – 2020	37 (68.5%)	39 (59.1%)	8 (20.5%)	24 (29.3%)	1 (6.3%)	5 (23.8%)
Fever > 38 C	54 (100%)	66 (100%)	39 (100%)	82 (100%)	16 (100%)	21 (100%)
-recurrent	28 (51.8%)	36 (54.5%)	N/A	N/A	9 (56.2%)	15 (71.4%)
-continued	7 (12.9%)	6 (9.1%)	N/A	N/A	6 (37.5%)	6 (28.6%)
Duration of the episode, days	6 (1—11)	4 (3—6)	2 (1—11)	4 (3—6)	6 (1—10)	4 (3—5)
Regular/ periodic attacks	52 (100%)	39 (59.1%)	22 (56.4%)	39 (47.6%)	N/A	N/A
Irregular/ non periodic attacks	0	21 (31.8%)	15 (38.5%)	42 (51.2%)	N/A	N/A
Generalized lymphadenopathy	0	1 (1.5%)	2 (5.1%)	8 (9.7%)	0	0
Cervical adenopathy	37 (68.5%)	31 (46.9%)	28 (71.7%)	39 (47.5%)	9 (56.2%)	6 (28.6%)
Muco-cutaneous signs						
•Aphthous stomatitis	11 (20.4%)	21 (31.8%)	21 (53.8%)	28 (34.1%)	5 (31.2%)	2 (9.5%)
Pharyngitis	43 (79.6%)	40 (60.6%)	14 (35.9%)	24 (29.2%)	3 (18.7%)	6 (28.6%)
Gastrointestinal involvement						
•Abdominal pain/fevers	20 (37%)	30 (45.4%)	10 (25.6%)	28 (34.1%)	5 (31.5%)	7 (33.3%)
•Vomitting	7 (12.9%)	11 (16.7%)	5 (12.8%)	13 (15.8%)	3 (18.7%)	0
Muscle-skeletal involvement						
Artralgia	16 (29.6%)	24 (36.4%)	6 (15.3%)	17 (20.7%)	3 (18.7%)	7 (33.3%)
Myalgia	0	1 (1.5%)	0	2 (2.4%)	0	4 (19%)
Morning stiffness	0	1 (1.5%)	0	0	1 (6.3%)	0
Other manifestations						
Headache	24 (44.4%)	21 (31.8%)	17 (43.5%)	19 (23.1%)	6 (37.5%)	5 (23.8%)
Hepatomegaly	1 (1.8%)	1 (1.5%)	1 (2.5%)	1 (1.2%)	0	1 (4.7%)
Pleuritis	0	0	0	0	0	3 (14.3%)
Pericarditis	0	0	0	0	0	2 (9.5%)

^a^Median, q1-q3

We observed that a subgroup of PFAPA patients, comprising up to 20% of the cohort, exhibited characteristics that aligned with the preliminary case definition for SURF. These patients demonstrated longer episodes of fever lasting between 2 to 6 days and a lower incidence of pharyngitis (ranging from 18.7% to 35.9%) based on data from AIDnet and Eurofever patients. Additionally, aphthous stomatitis was rare among these patients, ranging from 20.4% to 31.2% according to data from JIR-cohort and AIDnet. Importantly, this subgroup of PFAPA patients demonstrated a broader range of additional symptoms, such as abdominal pain (31.5% to 37%), vomiting (12.9% to 18.7%), arthralgia (18.7% 29.6%) and headache (37.5% to 44.4%).

**Fig. 3:**
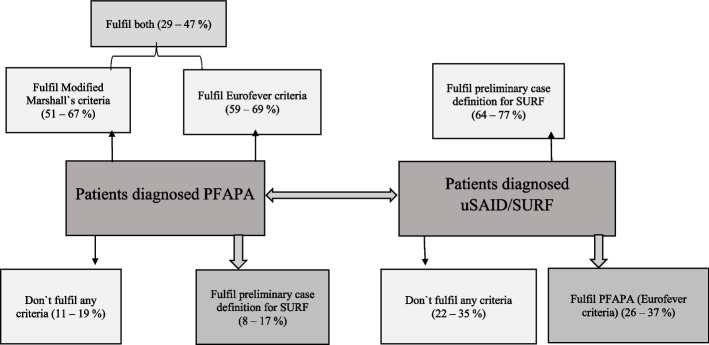
Final illustration of the patients diagnosed PFAPA and uSAID/SURF completing different criteria

It is noteworthy that a significant proportion, up to 38%, of uSAID/SURF patients met the Eurofever/PRINTO criteria for PFAPA (as shown in Table 4). These patients exhibited clinical symptoms commonly associated with PFAPA, including recurrent fever (54% to 71%), pharyngitis (28% to 60%), cervical adenopathy (28% to 47%) and aphthous stomatitis (9% to 34%). They also presented with arthralgia (33% to 36%), abdominal pain (33% to 45%) and headache (23% to 31%). These findings indicate the presence of a heterogeneous group of patients within both PFAPA and uSAID/SURF categories, with variable clinical presentations that do not strictly align them with either PFAPA or uSAID/SURF (refer to Fig. 3).

## Discussion

The management of pediatric or young adult patients with SAIDs poses significant challenges for healthcare professionals across multiple specialties, including paediatricians, rheumatologists, and adult clinical immunologists. Among the various autoinflammatory disorders, PFAPA syndrome stands out as a common and well-recognized clinical entity in the pediatric population, while hereditary fever disorders are less frequently diagnosed in the United States and Western Europe. Despite the existence of approved classification criteria for PFAPA, diagnosing this syndrome remains a considerable challenge. Since its initial description in 1987, PFAPA has often been misinterpreted as upper respiratory infections, leading to inappropriate treatment strategies, including unnecessary antibiotic prescriptions. The non-specific nature of PFAPA symptoms, such as recurrent fever, pharyngitis, and cervical adenopathy, complicates diagnosis, which is primarily based on the exclusion of other infectious and autoinflammatory diseases. As a result, definitive diagnosis is typically reserved for specialists with extensive experience in managing this patient population [14]. It is increasingly recognized that a subset of children presenting with non-infectious recurrent fever cannot be classified within existing diagnostic categories. These patients, often referred to as having uSAID or syndrome of SURF, pose unique challenges for clinical management. The current landscape of managing these patients is characterized by the need for updating definitions and classification categories to better characterize and understand this heterogeneous group [8, 12, 15, 16, 17]

The distinction between patients falling into the PFAPA category versus those categorized as uSAID/SURF presents a significant challenge, particularly when considering the clinical patterns and associated symptoms. While some patients may meet criteria for SURF due to variations in periodicity and symptomatology, they could alternatively be classified as PFAPA depending on the criteria used. Our study sheds light on this potential overlap and aims to elucidate the evolving nature of PFAPA and its relationship with uSAID/SURF. PFAPA patients that were evaluated through the registries performed incomplete response towards applied criteria, in which it demonstrated a separate group of non-responders that potentially may perform overlapping group with SURF (Fig. 3). PFAPA is a self-limiting disease that has a favourable prognosis. Usually, it resolves spontaneously before adolescence without impairment of growth and development, yet, with less knowledge about the long-term outcomes of this disease. There are very few data on the prognosis of PFAPA lasting up to adolescence and beyond [18–22].

The re-evaluation of the patients enrolled in the three registries as PFAPA and undefined recurrent fever according to the available PFAPA clinical criteria or case definition for SURF prompted rather heterogeneous results. In routine clinical practice, the diagnosis of PFAPA is based on summary of clinical judgements – with stereotypical attacks and prompt steroid response. Conversely uSAID/SURF should be defined by the absence of clinical manifestation typical for PFAPA in the absence of pathogenic mutations for genes associated with inherited recurrent fever, display a good response to colchicine treatment [23]. Globally up to 67% of patients enrolled as PFAPA fulfilled at least one PFAPA criteria only and up to 77% patients enrolled as undefined recurrent fever fulfilled the preliminary case definition for SURF only. Conversely, up to 17% of PFAPA and up to 37% of patients enrolled as undefined recurrent fever patients fulfilled at least one criteria associated to the alternative condition. Finally, up to 19% of PFAPA and 35% of SURF patients did not fulfil any clinical criteria.

The heterogeneity observed among patients diagnosed with PFAPA and SURF in the registries underscores the need for further evaluation and differentiation within these groups. This diversity in clinical presentation raises concerns regarding the potential for misdiagnosis and highlights the importance of refining diagnostic criteria to better characterize these conditions. The phenotypic heterogeneity observed in our study suggests that the current diagnostic labels may not fully capture the spectrum of presentations seen in patients with recurrent fever syndromes. As such, there is a pressing need for more precise classification criteria that can accurately distinguish between different subtypes of autoinflammatory diseases. This study clearly shows the existence of a large gray zone of overlap between these two conditions, at least looking into the retrospective data of three large international registries.

Our study has several limitations that should be acknowledged. Firstly, most of the patients were enrolled in the registries before the development and the publication of the evidence-based Eurofever PFAPA criteria and the proposed case definition for SURF. This issue clearly explains the high phenotypic variability observed in the study, since the patients were enrolled in the registries according to the clinical diagnosis proposed by the enrolling centres. It should also be noted that the proposed case definition for SURF by PAPA et al. was not developed with an evidence-based approach comparing different conditions but simply derived from the critical revision of the literature. Additionally, the lack of prospective follow-up data limits our ability to draw definitive conclusions regarding therapeutic response and long-term outcomes.

Another relevant limitation of the present study was the lack of extensive genetic analysis in the entire population of included patients. Hence, genetic diagnosis might have been missed, due to the absence of the relevant genes testing. Genetic screening was often limited in this particular group of the patients for following reasons: large geographical distribution of the enrolling centres and limited availability of molecular screening; enrolment of the patients since 2009 before the availability of Next Generation Sequencing and routine use of gene panels; real-life diagnosing of PFAPA without genetic testing, based exclusively on clinical criteria.

Despite these limitations it should be underlined that a relevant number of patients with undefined autoinflammatory disease does not find a genetic definition even after an extensive genetic analysis with whole exome or genome sequencing [24]. The relevant number of patients presenting signs of undefined recurrent fevers, even after genetic testing, requires further evaluation and follow-up, that generally suggests a need for further discoveries in the field [7, 25]. The most important point for the clinician is to distinguish self-limiting disease from long-term conditions with high risk of damage and significant impact on future well-being of the patient. The development of evidence-based classification criteria for SURF based on the comparison of the most frequent causes of recurrent fever (PFAPA and inherited recurrent fevers) could help to identify a more homogeneous subset of patients with undefined recurrent fever; the acronym of uSAID may be more appropriate for patients without the criteria of PFAPA. In any case, patients whose clinical presentation is uncertain, recommended to be followed with gene panels of next generation sequencing and whole genome sequencing in case if the first one is negative [26, 27]. We want to stress the importance of the thorough diagnostic of patients with undefined autoinflammatory phenotype and if necessary, re-evaluate them with a time new information and updates are available. Such results potentially may improve treatment outcomes, as far most of defined SAIDs present favourable response to steroids, colchicine or IL1-blocking therapy [28–30]. Contrary to the favourable effect observed in PFAPA to tonsillectomy, or aberrant effect of steroids, patients with undefined SAIDs rarely will perform the same response. But uSAID/SURF patients perform a favourable response to colchicine therapy, where in severe cases IL-1 therapy may dramatically resolve clinical presentation [31]. Early differentiation between these conditions will be beneficial for further follow-up management.

Further prospective studies are required to delineate fully the manifestations, diagnostic and treatment outcomes of children and adult patients with PFAPA and uSAID/SURF.

## Conclusion

The study provides insights into the clinical heterogeneity of undefined recurrent fever through the analysis of data from three European AID registries, that aimed to unravel the evolving symptomatology of patients with undifferentiated AIDs, focusing on PFAPA and SURF. Our findings underscore the complexity of diagnosing and classifying patients with recurrent fevers without a clear genetic etiology with a significant proportion of patients did not fit neatly into existing diagnostic criteria for PFAPA or SURF, highlighting the need for refined classification criteria and updated definitions. The study elucidated a spectrum of clinical presentations among patients diagnosed with PFAPA, SURF/uSAID and revealed overlaps in symptomatology between groups, suggesting potential misclassification and emphasizing the importance of accurate diagnosis for tailored treatment strategies.

Finally, this study underscores the necessity of refining diagnostic criteria, updating classification categories, and improving our understanding of the clinical heterogeneity of autoinflammatory diseases. By addressing these challenges, we can advance patient care, enhance prognostic accuracy, and facilitate the development of targeted therapeutic interventions for individuals with recurrent fevers of unknown origin.

### Supplementary Information

**Supplementary Material 1.**

## Data Availability

The datasets used and/or analyzed during the current study are available from the corresponding author upon reasonable request.

## Abbreviations

SAIDs: Systemic autoinflammatory disorders
AID: Autoinflammatory disease
PFAPA: Periodic fever, aphthous stomatitis, pharyngitis, and adenitis syndrome
uAID: Undefined autoinflammatory diseases
PRES: Pediatric Rheumatology European Society
SURF: Syndrome of undifferentiated recurrent fever
HRF: Hereditary recurrent fevers
ERN RITA: European Reference Network on Rare Primary Immunodeficiency, Autoinflammatory, and Autoimmune Diseases
AID: Net Network for Inflammatory Diseases

## References

[1] Hernández-Rodríguez J, Ruíz-Ortiz E, Tomé A, Espinosa G, González-Roca E, Mensa-Vilaró A, Prieto-González S, Espígol-Frigolé G, Mensa J, Cardellach F, Grau JM, Cid MC, Yagüe J, Aróstegui JI, Cervera R (2016). Clinical and genetic characterization of the autoinflammatory diseases diagnosed in an adult reference center. Autoimmun Rev.

[2] Rigante D, Vitale A, Lucherini OM, Cantarini L (2014). The hereditary autoinflammatory disorders uncovered. Autoimmun Rev.

[3] Førsvoll J, Kristoffersen EK, Øymar K (2013). Incidence, clinical characteristics and outcome in Norwegian children with periodic fever, aphthous stomatitis, pharyngitis and cervical adenitis syndrome; a population-based study. Acta Paediatr.

[4] Hofer M, Pillet P, Cochard MM, Berg S, Krol P, Kone-Paut I, Rigante D, Hentgen V, Anton J, Brik R, Neven B, Touitou I, Kaiser D, Duquesne A, Wouters C, Gattorno M (2014). International periodic fever, aphthous stomatitis, pharyngitis, cervical adenitis syndrome cohort: description of distinct phenotypes in 301 patients. Rheumatology.

[5] Vanoni F, Caorsi R, Aeby S, Cochard M, Antón J, Berg S, Brik R, Dolezalova P, Koné-Paut I, Neven B, Ozen S, Pillet P, Stojanov S, Wouters C, Gattorno M, Hofer M (2018). Towards a new set of classification criteria for PFAPA syndrome. Pediatr Rheumatol Online J.

[6] Vanoni F, Federici S, Antón J, Barron KS, Brogan P, De Benedetti F, Dedeoglu F, Demirkaya E, Hentgen V, Kallinich T, Laxer R, Russo R, Toplak N, Uziel Y, Martini A, Ruperto N, Gattorno M, Hofer M; for Eurofever and the Paediatric Rheumatology International Trials Organisation (PRINTO). An international delphi survey for the definition of the variables for the development of new classification criteria for periodic fever aphtous stomatitis pharingitis cervical adenitis (PFAPA). Pediatr Rheumatol Online J. 2018 Apr 18;16(1):27. doi: 10.1186/s12969-018-0246-9. PMID: 29669569; PMCID: PMC5907175. 10.1186/s12969-018-0246-9 PMC5907175 29669569

[7] Adrovic A, Yıldız M, Kanber M, Ulkersoy I, Gucuyener N, Koker O, Sahin S, Barut K, Kasapcopur O (2020). Performance of recently proposed periodic fever, aphthous stomatitis, pharyngitis, and cervical adenitis (PFAPA) syndrome criteria in a region endemic for familial Mediterranean fever. Rheumatol Int.

[8] Ter Haar NM, Eijkelboom C, Cantarini L, Papa R, Brogan PA, Kone-Paut I, Modesto C, Hofer M, Iagaru N, Fingerhutová S, Insalaco A, Licciardi F, Uziel Y, Jelusic M, Nikishina I, Nielsen S, Papadopoulou-Alataki E, Olivieri AN, Cimaz R, Susic G, Stanevica V, van Gijn M, Vitale A, Ruperto N, Frenkel J, Gattorno M; Eurofever registry and the Pediatric Rheumatology International Trial Organization (PRINTO). Clinical characteristics and genetic analyses of 187 patients with undefined autoinflammatory diseases. Ann Rheum Dis. 2019 Oct;78(10):1405–1411. doi: 10.1136/annrheumdis-2018-214472. Epub 2019 Jul 5. PMID: 31278138. 10.1136/annrheumdis-2018-214472 31278138

[9] De Pauli S, Lega S, Pastore S, Grasso DL, Bianco AMR, Severini GM, Tommasini A, Taddio A (2018). Neither hereditary periodic fever nor periodic fever, aphthae, pharingitis, adenitis: Undifferentiated periodic fever in a tertiary pediatric center. World J Clin Pediatr.

[10] Ozen S, Frenkel J, Ruperto N, Gattorno M; Eurofever Project. The Eurofever Project: towards better care for autoinflammatory diseases. Eur J Pediatr. 2011 Apr;170(4):445–52. doi: 10.1007/s00431-011-1411-z. Epub 2011 Mar 1. PMID: 21360011. 10.1007/s00431-011-1411-z 21360011

[11] Toplak N, Frenkel J, Ozen S, Lachmann HJ, Woo P, Koné-Paut I, De Benedetti F, Neven B, Hofer M, Dolezalova P, Kümmerle-Deschner J, Touitou I, Hentgen V, Simon A, Girschick H, Rose C, Wouters C, Vesely R, Arostegui J, Stojanov S, Ozgodan H, Martini A, Ruperto N, Gattorno M; Paediatric Rheumatology International Trials Organisation (PRINTO), Eurotraps and Eurofever Projects. An international registry on autoinflammatory diseases: the Eurofever experience. Ann Rheum Dis. 2012 Jul;71(7):1177–82. doi: 10.1136/annrheumdis-2011-200549. Epub 2012 Feb 29. PMID: 22377804. 10.1136/annrheumdis-2011-200549 22377804

[12] Papa R, Penco F, Volpi S, Sutera D, Caorsi R, Gattorno M (2021). Syndrome of Undifferentiated Recurrent Fever (SURF): An Emerging Group of Autoinflammatory Recurrent Fevers. J Clin Med.

[13] Vyzhga Y, Hentgen V, Caorsi R, Wittkowski H, Hofer M, Ruperto N, Lainka E, Theodoropoulou K, Foell D, Mosci E, Gattorno M; AID-Net, Eurofever, JIRcohort registriesthe Paediatric Rheumatology International Trials Organisation (PRINTO). Breaking down the fences among registries on autoinflammatory diseases: the E-Merge project. Orphanet J Rare Dis. 2023 Jul 17;18(1):191. doi: 10.1186/s13023-023-02812-4. PMID: 37461074; PMCID: PMC10353236. 10.1186/s13023-023-02812-4 PMC10353236 37461074

[14] Rusmini M, Federici S, Caroli F, Grossi A, Baldi M, Obici L, Insalaco A, Tommasini A, Caorsi R, Gallo E (2016). Next-Generation Sequencing and Its Initial Applications for Molecular Diagnosis of Systemic Auto-Inflammatory Diseases. Ann Rheum Dis.

[15] Amarilyo G, Rothman D, Manthiram K, Edwards KM, Li SC, Marshall GS, Yildirim-Toruner C, Haines K, Ferguson PJ, Lionetti G, Cherian J, Zhao Y, DeLaMora P, Syverson G, Nativ S, Twilt M, Michelow IC, Stepanovskiy Y, Thatayatikom A, Harel L, Akoghlanian S, Tucker L, Marques MC, Srinivasalu H, Propst EJ, Licameli GR, Dedeoglu F, Lapidus S; CARRA PFAPA Consensus Treatment Plan Workgroup. Consensus treatment plans for periodic fever, aphthous stomatitis, pharyngitis and adenitis syndrome (PFAPA): a framework to evaluate treatment responses from the childhood arthritis and rheumatology research alliance (CARRA) PFAPA work group. Pediatr Rheumatol Online J. 2020 Apr 15;18(1):31. doi: 10.1186/s12969-020-00424-x. PMID: 32293478; PMCID: PMC7157990. 10.1186/s12969-020-00424-x PMC7157990 32293478

[16] Gattorno M, Hofer M, Federici S, Vanoni F, Bovis F, Aksentijevich I, Anton J, Arostegui JI, Barron K, Ben-Cherit E, Brogan PA, Cantarini L, Ceccherini I, De Benedetti F, Dedeoglu F, Demirkaya E, Frenkel J, Goldbach-Mansky R, Gul A, Hentgen V, Hoffman H, Kallinich T, Kone-Paut I, Kuemmerle-Deschner J, Lachmann HJ, Laxer RM, Livneh A, Obici L, Ozen S, Rowczenio D, Russo R, Shinar Y, Simon A, Toplak N, Touitou I, Uziel Y, van Gijn M, Foell D, Garassino C, Kastner D, Martini A, Sormani MP, Ruperto N; Eurofever Registry and the Paediatric Rheumatology International Trials Organisation (PRINTO). Classification criteria for autoinflammatory recurrent fevers. Ann Rheum Dis. 2019 Aug;78(8):1025–1032. doi: 10.1136/annrheumdis-2019-215048. Epub 2019 Apr 24. PMID: 31018962. 10.1136/annrheumdis-2019-215048 31018962

[17] Broderick L, Hoffman HM. Pediatric recurrent fever and autoinflammation from the perspective of an allergist/immunologist. J Allergy Clin Immunol. 2020 Nov;146(5):960–966.e2. doi: 10.1016/j.jaci.2020.09.019. Epub 2020 Sep 28. PMID: 33002514; PMCID: PMC8559310. 10.1016/j.jaci.2020.09.019 PMC8559310 33002514

[18] Fayand A, Hentgen V, Ducharme-Bénard S, Quartier P, Bader-Meunier B, Koné-Paut I, Grateau G, Georgin-Lavialle S. Do we need the PFAPA syndrome in adults with non-monogenic periodic fevers? Ann Rheum Dis. 2022 Jan;81(1):e15. doi: 10.1136/annrheumdis-2019-216827. Epub 2019 Dec 31. PMID: 31892530. 10.1136/annrheumdis-2019-216827 31892530

[19] Vitale A, Orlando I, Lopalco G, Emmi G, Cattalini M, Frediani B, Galeazzi M, Iannone F, Rigante D, Cantarini L. Demographic, clinical and therapeutic findings in a monocentric cohort of adult patients with suspected PFAPA syndrome. Clin Exp Rheumatol. 2016 Sep-Oct;34(6 Suppl 102):77–81. Epub 2016 Oct 25. PMID: 27791949. 27791949

[20] Cantarini L, Vitale A, Bartolomei B, Galeazzi M, Rigante D. Diagnosis of PFAPA syndrome applied to a cohort of 17 adults with unexplained recurrent fevers. Clin Exp Rheumatol. 2012 Mar-Apr;30(2):269–71. Epub 2012 Apr 13. PMID: 22325152. 22325152

[21] Cantarini L, Vitale A, Sicignano LL, Emmi G, Verrecchia E, Patisso I, Cerrito L, Fabiani C, Cevenini G, Frediani B, Galeazzi M, Rigante D, Manna R (2017). Diagnostic Criteria for Adult-Onset Periodic Fever, Aphthous Stomatitis, Pharyngitis, and Cervical Adenitis (PFAPA) Syndrome. Front Immunol.

[22] Poker Y, von Hardenberg S, Hofmann W, Tang M, Baumann U, Schwerk N, Wetzke M, Lindenthal V, Auber B, Schlegelberger B, Ott H, von Bismarck P, Viemann D, Dressler F, Klemann C, Bergmann AK (2023). Systematic genetic analysis of pediatric patients with autoinflammatory diseases. Front Genet.

[23] Welzel T, Ellinghaus M, Wildermuth AL, Deschner N, Benseler SM, Kuemmerle-Deschner JB (2021). Colchicine Effectiveness and Safety in Periodic Fever, Aphthous Stomatitis, Pharyngitis, and Adenitis. Front Pediatr.

[24] Batlle-Masó L, Mensa-Vilaró A, Solís-Moruno M, Marquès-Bonet T, Arostegui JI, Casals F. Genetic diagnosis of autoinflammatory disease patients using clinical exome sequencing. Eur J Med Genet. 2020 May;63(5):103920. doi: 10.1016/j.ejmg.2020.103920. Epub 2020 Mar 25. PMID: 32222431. 10.1016/j.ejmg.2020.103920 32222431

[25] Banday AZ, Joshi V, Arora K, Sadanand R, Basu S, Pilania RK, Jindal AK, Vignesh P, Gupta A, Sharma S, Dhaliwal M, Rawat A, Singh S, Suri D (2022). Challenges in the diagnosis of periodic fever, aphthous stomatitis, pharyngitis, and adenitis syndrome in developing countries-A decade of experience from North India. Front Immunol.

[26] Sözeri B, Demir F, Sönmez HE, Karadağ ŞG, Demirkol YK, Doğan ÖA, Doğanay HL, Ayaz NA (2021). Comparison of the clinical diagnostic criteria and the results of the next-generation sequence gene panel in patients with monogenic systemic autoinflammatory diseases. Clin Rheumatol.

[27] Demir F, Doğan ÖA, Demirkol YK, Tekkuş KE, Canbek S, Karadağ ŞG, Sönmez HE, Ayaz NA, Doğanay HL, Sözeri B (2020). Genetic panel screening in patients with clinically unclassified systemic autoinflammatory diseases. Clin Rheumatol.

[28] Marques MC, Egeli BH, Wobma H, Ribeiro C, Anderson E, Hausmann JS, Dedeoğlu F (2022). Features predicting colchicine efficacy in treatment of children with undefined systemic autoinflammatory disease: A retrospective cohort study. Eur J Rheumatol.

[29] Luu I, Nation J, Page N, Carvalho D, Magit A, Jiang W, Leuin S, Bliss M, Bothwell M, Brigger M, Kearns D, Pransky S, Broderick L. Undifferentiated recurrent fevers in pediatrics are clinically distinct from PFAPA syndrome but retain an IL-1 signature. Clin Immunol. 2021 May;226:108697. doi: 10.1016/j.clim.2021.108697. Epub 2021 Feb 24. PMID: 33636366; PMCID: PMC8089050. 10.1016/j.clim.2021.108697 PMC8089050 33636366

[30] Papa R, Rusmini M, Volpi S, Caorsi R, Picco P, Grossi A, Caroli F, Bovis F, Musso V, Obici L, Castana C, Ravelli A, Van Gijn ME, Ceccherini I, Gattorno M (2020). Next generation sequencing panel in undifferentiated autoinflammatory diseases identifies patients with colchicine-responder recurrent fevers. Rheumatology (Oxford).

[31] Sutera D, Bustaffa M, Papa R, Matucci-Cerinic C, Matarese S, D'Orsi C, Penco F, Prigione I, Palmeri S, Bovis F, Volpi S, Caorsi R, Gattorno M. Clinical characterization, long-term follow-up, and response to treatment of patients with syndrome of undifferentiated recurrent fever (SURF). Semin Arthritis Rheum. 2022 Aug;55:152024. doi: 10.1016/j.semarthrit.2022.152024. Epub 2022 May 10. Erratum in: Semin Arthritis Rheum. 2023 Jun;60:152195. PMID: 35598507. 10.1016/j.semarthrit.2022.152024 35598507

