# Supplementary material for: Unravelling the clinical heterogeneity of undefined recurrent fever over time in the European registries on Autoinflammation
Source: Pediatr Rheumatol Online J. 2024 May 17;22:55. doi: 10.1186/s12969-024-00987-z (PMC11100049; doi:10.1186/s12969-024-00987-z)
Supplement: Supplementary file 1 — Supplementary Material 1. [file 12969_2024_987_MOESM1_ESM.docx]

Supplementary Table 1. Set of the clinical classification criteria used in the group of PFAPA patients among registries.

| Modified Marshall`s criteria | Eurofever/PRINTO clinical  classification criteria |
| --- | --- |
| Regularly recurring fevers with an early age onset (< 5 years of age)  Constitutional symptoms in absence of upper respiratory infection with at least one of the following clinical signs:  • aphthous stomatitis  • cervical lymphadenitis  • pharyngitis  Exclusion of cyclic neutropenia  Completely asymptomatic interval between episodes  Normal growth and development | At least seven out of eight: Presence   - Pharyngotonsillitis. - Duration of episodes, 3–6 days. - Cervical lymphadenitis. - Periodicity.   Absence   - Diarrhea. - Chest pain. - Skin rash. - Arthritis. |

Supplementary Table 2. Epidemiological and clinical data of the patients with PFAPA

|  | JIR-cohort (n = 317) | | | | Eurofever (n = 476) | | | | AID-net (n = 136) | | | |
| --- | --- | --- | --- | --- | --- | --- | --- | --- | --- | --- | --- | --- |
|  | Fulfil  Marshall`s criteria  (n =174) | Fulfil  Eurofever criteria  (n =220) | Fulfil  Both  (n = 122) | Don`t  fulfil any  (n = 45) | Fulfil Marshall`s criteria  (n =320) | Fulfil  Eurofever criteria  (n = 330) | Fulfil both  (n = 226) | Don`t fulfil any (n = 52) | Fulfil  Marshall`s criteria  (n = 70) | Fulfil  Eurofever criteria  (n = 80) | Fulfil both  (n = 40) | Don`t fulfil any  (n = 26) |
| Age of diagnose, years** | 3.16  (0.81 – 4.99) | 4.43*  (0.81 – 44.5) | 3.91  (0.81 – 4.99) | 4.23  (0.98 – 11.8) | 3.0  (1.2 – 4.58) | 3.7*  (2.1 – 9.1) | 3.4  (1.2 – 4.83) | 3.9  (1.6 – 9.4) | 3.0  (0.75 - 5) | 4.2*  (1 – 15.6) | 3.7  (0.75 - 5) | 4.36  (1.1 – 9.8) |
| Diagnostic delay, years** | 1.6  (0.1 – 5.02) | 2.21  (0.1 – 40.9) | 1.72  (0.1 – 4.67) | 2.23  (0.4 – 11.2) | 1.18  (0.1 – 2.8) | 1.41  (0.4 – 5.4) | 1.29  (0.1 – 3.6) | 1.62  (0.4 – 7.2) | 1.2  (0.16 – 4.16) | 1.58  (0.08 – 8.6) | 1.26  (0.1 - 4.4) | 1.6  (0.2 – 8.7) |
| Boys/girls | 90/84 | 126/88 | 68/54 | 28/17 | 163/157 | 190/140 | 117/109 | 28/24 | 39/31 | 46/34 | 21/19 | 15/11 |
| Patients <18 y.o. when diagnosed  Patients >18 y.o. when diagnosed | 174 (100%)  0 | 204 (92.7 %)  16 (7.3 %) | 122 (100%)  0 | 41 (91.1 %)  4 (8.9 %) | 320 (100%)  0 | 330 (100%)  0 | 226 (100%)  0 | 52 (100%)  0 | 70 (100%)  0 | 80 (100%)  0 | 40 (100%)  0 | 26 (100%)  0 |
| Enrolled in time-period:   - Under 2009 - 2009 – 2012 - 2013 – 2016 - 2017 – 2020 | N/A  N/A  77 (44.3 %)  97 (55.7 %) | N/A  2 (0.9 %)  111 (50.5 %)  107 (48.6 %) | N/A  0  60 (49.2 %)  62 (50.8 %) | N/A  N/A  21 (46.7 %)  24 (53.3 %) | 91 (28.4%)  141 (44.1%)  32 (10 %)  56 (17.5 %) | 98 (29.7 %)  130 (39.4%)  42 (12.7 %)  60 (18.2 %) | 63 (27.8 %)  88 (38.9 %)  27 (11.9 %)  48 (21.2 %) | 12 (23.1 %)  17 (32.7 %)  13 (25 %)  10 (19.2 %) | N/A  15 (21.4 %)  29 (41.4 %)  26 (37.1 %) | N/A  17 (21.3 %)  34 (42.5 %)  29 (36.2 %) | N/A  9 (22.5 %)  18 (45 %)  13 (32.5 %) | N/A  5 (19.2 %)  11 (42.3%)  10 (38.4 %) |
| Fever >38 C  Duration of the episode, days | 174 (100 %)  4 (1 - 6) | 220 (100 %)  4 (3 - 5) | 122 (100%)  4 (3 - 6) | 39 (86.7 %)  4 (1 - 11) | 320 (100 %)  3 (0.5 – 6.0) | 330 (100 %)  3 (3.0 – 6.0) | 226 (100%)  3 (3 - 6) | 44 (84.6 %)  4 (1 - 9) | 70 (100 %)  3 (1.0 – 6.0) | 80 (100 %)  3 (3.0 – 6.0) | 40 (100%)  3 (3 - 6) | 26 (100%)  4 (2 - 7) |
| Pharyngitis | 134 (77 %) | 173 (78.6 %) | 113 (92.6%) | 28 (62.2 %) | 211 (65.9%) | 291 (88.2%) | 173 (76.5%) | 36 (69.2%) | 41 (58.6%) | 54 (67.5 %) | 31 (77.5%) | 8 (30.7 %) |
| Cervical adenopathy | 107 (61.5 %) | 149 (67.7 %) | 92 (75.4 %) | 23 (51.1 %) | 219 (68.4%) | 243 (73.6%) | 159 (70.3%) | 34 (65.3%) | 44 (62.8 %) | 61 (76.2 %) | 34 (85%) | 11 (42.3 %) |
| Aphthous stomatitis | 61 (35 %) | 91 (41.3 %) | 49 (40.1 %) | 16 (35.5 %) | 139 (43.4 %) | 203 (61.5 %) | 106 (46.9 %) | 21 (40.4 %) | 27 (38.6 %) | 35 (43.7 %) | 21 (52.5 %) | 8 (30.7 %) |
| Others  Headache  Diarrhoea  Rash  Arthritis | 29 (16.7 %)  24 (13.8 %)  6 (3.4 %)  3 (1.7 %) | 58 (26.4 %)  0  0  0 | 22 (18 %)  0  0  0 | 17 (37.8 %)  19 (42.2%)  13 (28.9 %)  3 (6.7 %) | 47 (14.7 %)  27 (8.6 %)  20 (6.3 %)  5 (1.6 %) | 68 (20.6 %)  0  0  0 | 35 (15.5 %)  0  0  0 | 14 (26.9 %)  36 (69.2%)  17 (32.6%)  4 (7.7%) | 17 (24.3 %)  12 (17.1 %)  6 (8.6 %)  0 | 21 (26.2 %)  0  0  0 | 15 (37.5 %)  0  0  0 | 9 (34.6 %)  10 (38.5 %)  4 (15.4 %)  2 (7.7 %) |

*p<0.05 – to compare with PFAPA cases that fulfil modified Marshall`s criteria

**Median, q1-q3

Approach of the application of modified Marshal criteria and Eurofever/PRINTO criteria was first performed on general cohort of selected PFAPA patients (presented in column 1 and 2 of each registry set). Than combination of the criteria and broad description of the rest patients, who formally not completed none of the above (column 3 and 4 of each registry set).
